# Is heavy eccentric calf training superior to wait-and-see, sham rehabilitation, traditional physiotherapy and other exercise interventions for pain and function in mid-portion Achilles tendinopathy?

**DOI:** 10.1186/s13643-018-0725-6

**Published:** 2018-04-13

**Authors:** Myles Murphy, Mervyn Travers, William Gibson

**Affiliations:** 1 School of Physiotherapy, The University of Notre Dame Australia, 19 Mouat Street, PO Box 1225, Fremantle, Western Australia 6959 Australia; 2 SportsMed Subiaco, St John of God Health Care, Subiaco, Australia; 3 Sports Science Sports Medicine Department, Western Australian Cricket Association, East Perth, Australia; 4 School of Physiotherapy and Exercise Science, Curtin University, Bentley, Australia

**Keywords:** Achilles, Tendinopathy, Heel pain, Tendinitis, Systematic review, Meta-analysis, Alfredson

## Abstract

**Background:**

Mid-portion Achilles tendinopathy (AT) is prevalent amongst athletic and non-athletic populations with pain, stiffness and impaired function typically reported. While different management options exist, loading protocols remain the best available intervention and have been shown to be effective in the management of AT. Trials investigating loading in AT have used a variety of different protocols, and recent narrative reviews suggest that no protocol is superior to another when comparing outcomes in pain and function. However, there has been no systematic review or meta-analysis completed to determine this. Furthermore, the narrative review did not consider wait-and-see or sham interventions, thus a systematic review and met-analysis which includes wait-and-see or sham interventions is warranted.

**Methods:**

A systematic review and meta-analyses will be conducted as per the PRISMA guidelines. The databases PUBMED, CINAHL (Ovid) and CINAHL (EBSCO) will be searched for articles published from inception to 31 December 2017. Our search focuses on studies examining the improvement of pain and function when completing a loading program for mid-portion AT. Only randomised/ quasi-randomised trials will be included while case reports and case series will be excluded. The primary outcome assessing pain and function will be the Victorian Institute Sports Assessment - Achilles (VISA-A). Two reviewers will screen articles, extract data and assess the risk of bias independently with a third reviewer resolving any disagreements between the two reviewers. A meta-analysis will then be performed on the data (if appropriate) to determine if the traditional heavy load calf training protocol described by Alfredson is superior to wait-and-see, sham intervention, traditional physiotherapy, and other forms of exercise rehabilitation.

**Discussion:**

This systematic review and meta-analysis will allow us to investigate if there are difference in pain and function when comparing wait-and-see, sham interventions, traditional physiotherapy and different exercise interventions to the traditional heavy eccentric calf training protocol for mid-portion Achilles tendon pain.

**Systematic review registration:**

PROSPERO registration number CRD42018084493.

**Electronic supplementary material:**

The online version of this article (10.1186/s13643-018-0725-6) contains supplementary material, which is available to authorized users.

## Background

### Description of the condition

Clinically, tendinopathy is characterised by focal pain, morning stiffness, and restricted function [1]. Many different models have been presented to attempt to explain the link between tendon pain, function and structure. However, pain is complex, and there is evidence both for and against a relationship between pain, function and tendon structure [2]. To date, three different models are used to describe tendinopathy; collagen disruption/tearing, tendon cell response and inflammation. The continuum of tendinopathy is used to help describe tendon pain in relation to pathology [1, 2]. The collagen disruption/ tearing models suggests that tendon pain is caused from a catabolic response of the tendon cells due to a lack of loading caused secondary to microscopic collagen damage [3]. The tendon cell response model proposes that in response to loading, tenocytes will stimulate a response which in turn modifies the extracellular matrix [4]. The inflammatory model suggests that, in response to loading, an inflammatory response occurs which may relate to tendon degradation and disorganisation [5–7]. The continuum of tendinopathy presents a continuum of change from an acute reactive tendon to a chronic degenerative tendon [1, 2].

### Prevalence of the condition

Achilles tendinopathy (AT) is prevalent within both the athletic (prevalence of 6.2–9.5%) [8] and non-athletic populations (prevalence of 11.83 per 100 patient years) [9].

### Description of the intervention

Tendinopathy is commonly treated with loading protocols, and these have been suggested to be highly effective in the management of this condition [10]. To date, in trials investigating loading in AT, four different loading protocols have been reported; heavy eccentric training, [11] concentric training, [12] eccentric overload training [13, 14], and heavy slow resistance training, [15] Recently, these loading protocols have been discussed in a narrative review, and the suggestion made that no protocol is superior to another when comparing outcomes in pain and function [10]. However, this has yet to be examined under systematic review methodology, and formal comparisons of the differing loading protocols against wait-and-see or sham rehabilitation have not been made. In 2009, a systematic review looked at determining the ideal dosage for eccentric training when rehabilitating mid-portion Achilles tendinopathy and was unable to make firm conclusions based on the research at the time [16]. Finally, in 2015 a systematic review investigating different protocols for eccentric training for mid-portion AT and found no difference between protocols [17]. Again, however, a wait-and-see or sham rehabilitation comparison was not undertaken.

### Why is it important to do this review?

This review is important as it is currently reported and widely accepted that exercise rehabilitation, specifically heavy load eccentric calf training, is the gold standard for rehabilitation of mid-portion AT. This approach has been accepted and promulgated in the absence of any systematic review comparing findings with wait-and-see or sham intervention groups. The other important aspect of this review is that heavy eccentric calf training is time consuming and if rehabilitation protocols which are less time consuming and have similar functional outcomes, this may change clinical practice.

### Objectives

To determine if heavy eccentric calf training is more effective than wait-and-see, sham interventions, traditional physiotherapy and other exercise rehabilitation protocols for improvements in pain and function in mid-portion Achilles tendinopathy.

## Methods

### Data management

Data will be managed and stored via The University of Notre Dame Australia’s online file server and OneDrive.

### Criteria for considering studies for this review

#### Types of studies

Randomised and quasi-randomised trials will be included if one study arm used heavy eccentric calf training to treat mid-portion AT and the other used a wait-and-see, sham rehabilitation or exercise intervention. Studies that have used an exercise intervention with a sham/ placebo group will be included (e.g. one arm of the study used heavy eccentric calf training with sham ultrasound and another arm used a modified version of heavy eccentric calf training with sham ultrasound). Identified studies regardless of their publication status will be included. Trials that are non-randomised observational trials, case reports/ series, clinical observations and systematic reviews will be excluded.

#### Types of participants

We will include both physically active and sedentary participants aged 18 years and over identified as having mid-portion AT for greater than three months. Studies that include participants with insertion Achilles tendinopathy or other cause of heel pain will be excluded from the review.

#### Types of wait-and-see and placebo

Wait-and-see groups will be included if they have no form of active or passive intervention. Sham intervention groups will only be classified as such if the participants underwent sham exercise interventions highly unlikely to impact participant pain and function. Specifically, exercise interventions that do not overload contractile tissue and are unlikely to induce a strength response will be classified as sham rehabilitation. For example, participants who underwent a calf stretching program or balance training program would be included into sham rehabilitation.

#### Types of traditional physiotherapy

Traditional physiotherapy groups will be included if the participants had no exercise intervention, either a real or sham exercise intervention, but had some form of traditional physiotherapy intervention. The traditional physiotherapeutic interventions included will be the following:

- Deep friction massage to the tendon, and/or
- Other forms of manual physical therapy to local tissues, and/or
- Ultrasound, and/or
- Strapping tape

Given the lack of robust evidence confirming that these interventions are not effective, and that there is also a lack of robust evidence saying these interventions may not interfere with an exercise rehabilitation protocol these interventions were not classified as sham.

#### Types of interventions

Intervention studies using either isometric, eccentric, concentric or isotonic (eccentric and concentric) loading protocols will be included. Also included are studies that have had an isometric, eccentric, concentric or isotonic loading program in conjunction with a placebo therapy (for example, sham laser treatment) as long as the comparator group within the same study underwent an identical placebo therapy. Comparisons of interest are the following:

- Heavy eccentric calf training versus wait-and-see
- Heavy eccentric calf training versus sham exercise
- Heavy eccentric calf training versus traditional physiotherapy
- Heavy eccentric calf training versus different exercise protocols

#### Types of outcomes measures

Only studies that used a validated and reliable outcome measure of pain and function in mid-portion Achilles tendinopathy will be included. A recent consensus statement recognised the Victorian Institute of Sports Assessment-Achilles (VISA-A) as a valid and reliable tool for assessing AT [18]. The VISA-A is a self-reported outcome measure which includes a variety of questions about both pain and function [19]. The VISA-A has also been identified as the only reliable and valid measure of pain and function in the mid-portion Achilles [20].

Trials which used the visual analogue scale (VAS) or numerical rating scale of pain will not be included. The VAS at rest has been shown to have a test-retest reliability of *r* = 0.45 in mid-portion Achilles tendinopathy [14]. The relationship between pain and function is also intricately linked in tendinopathy given symptoms are load dependant [1]. Therefore, including a measure of pain without linking it to function may introduce bias.

### Search methods for identification of studies

Search strategies will be implemented from inception until the 31 December 2017.

#### Electronic searches

Searches using free text terms (see Table 1) to identify published articles on the following electronic databases:

- PubMed
- CINAHL (Ovid);
- CINAHL (EBSCO)

**Table 1:** Systematic Review Search Strategy

Number	Combiners	Terms
1	Problem of interest	Achil* OR triceps surae* OR tend* OR heel OR calcan*
2	Intervention	Exercise OR eccentric* OR isotonic* OR heavy slow resistance OR isometrics OR resistance OR strength* OR alfredson*
3		#1 AND #2
4	Outcome	VISA* OR Victorian institute of sport score*
5		#3 AND #4
	Limitations	Peer reviewed, human, clinical trials

Only peer reviewed, human, clinical trials will be included; however, this will be adapted to individual databases as necessary. We will not restrict the language of publication to English.

#### Searching other resources

Additional searches will be conducted on the Cochrane Central Register of Controlled Trials, metaRegister of controlled trials (mRCT) (https://www.isrctn.com/), clinicaltrials.gov (www.clinicaltrials.gov) and the World Health Organization (WHO) International Clinical Trials Registry Platform (ICTRP) (apps.who.int/trialsearch/) for ongoing trials.

Reference lists for reviews and retrieved articles for additional studies will be checked, and citation searches on key articles performed. Experts in the field for unpublished and ongoing trials will be contacted. The list of included studies will be evaluated by content experts to help identify any additional relevant studies. Web of Science will also be used for forward citation tracking.

The ePublication lists of key journals in the field will be screened in an attempt to pick up studies which have yet to be indexed in the databases. The journals searched will include the following:

- British Journal of Sports Medicine
- Sports Medicine
- American Journal of Sports Medicine
- Medicine and Science in Sports and Exercise
- Journal of Science and Medicine in Sport
- Journal of Athletic Training
- Journal of Physiotherapy
- Archives of Physical Medicine and Rehabilitation
- Journal of Sports Sciences
- Scandinavian Journal of Medicine and Science in Sports
- Journal of Orthopaedic and Sports Physical Therapy
- Physical Therapy in Sport
- Physical Therapy
- Clinical Rehabilitation
- Clinical Journal of Sport Medicine
- Journal of Sports Rehabilitation
- Physiotherapy
- International Journal of Sports Physical Therapy

#### Unpublished data

In order to minimise the prospect of publication bias, a further search of the following will be undertaken:

- OpenGrey (System for Information on Grey Literature in Europe)
- Dissertation Abstracts (Proquest)
- SPORTDiscus

### Data collection and analysis

#### Selection of studies

Two review authors (MM and MT) will independently assess the titles and abstracts of potential studies identified by the search strategy for their eligibility. If the eligibility of a study is unclear from the title and abstract, the full paper will be assessed. Studies that do not match the inclusion criteria for this review will be excluded. Disagreements between authors regarding study inclusion will be resolved by discussion. A third review author (WG) will assess relevant studies if resolution and agreement cannot be reached and a majority decision made. Studies will not be anonymised prior to assessment.

A PRISMA study flow diagram [21] will be included in the full review to document the screening process as recommended in Part 2, Section 11.2.1 of the Cochrane Handbook for Systematic Reviews of Interventions [22].

#### Data extraction and management

Two review authors (MM and MT) will independently abstract data from all included studies using a standardised and piloted data extraction form. Discrepancies and disagreements will be resolved by consensus. In cases where consensus is not achieved, a third review author (WG) will assess the trial for arbitration with a majority decision. The following information from the review will be extracted:

- Primary author
- Year of publication
- Study design
- Study population (diagnosis)
- Sample size (including sample size at all follow-up points)
- Baseline demographics (age, height, weight, BMI, gender, duration of pain and country of study)
- Loading intervention
- Adherence to loading intervention
- Whether a placebo treatment was administered in conjunction with the loading intervention and characteristics of the control
- Concomitant treatments
- Mean (SD) of the VISA-A at baseline and the final follow-up time points while undergoing exercise loading.
- Time (weeks) at each follow-up point

The primary author, year of publication, study design, sample size, mean age (SD), mean pain duration (SD), the number of male and females, loading intervention and mean change from baseline of the VISA-A will be presented in a summary of findings table.

#### Assessment of risk of bias in included studies

Two review authors (MM and MT) will independently assess risk of bias for each study. Any disagreements will be resolved by discussion however in the event consensus cannot be achieved a third review author (WG) will make a majority decision.

Randomised trials will be assessed using the Cochrane Risk of Bias (RoB 2.0) tool and involves judgement on seven domains (http://www.riskofbias.info). Judgements on the risk of bias for each of the domains and overall risk of bias will be made as per the recommendations of the RoB 2.0 tool (Additional file 1). Trials will be classified overall as having either a low risk of bias, some concerns of bias or high risk of bias as described in the RoB 2.0 tool.

#### Measure of treatment effect

Primary outcomes will be presented and analysed on a continuous scale as mean difference with 95% confidence intervals given the same scale (VISA-A) will be used across all trials. Data will be analysed at the final time point while the patient is undergoing the loading intervention.

#### Dealing with missing data

Where insufficient data is presented in the study report to enter into meta-analysis study authors will be contacted to request access to the missing data. Trials where data cannot be accessed following contact with the author will be excluded from the meta-analyses.

If the trial does not provide the standard deviation and after contacting the authors, they do not provide the standard deviation, the standard deviation will be imputed from another trial which has used the same outcome measure (VISA-A) at an identical follow-up time point. This method is recommended in Part 3, Section 16.1.3.2 of the Cochrane Handbook for Systematic Reviews of Interventions [22].

#### Assessment of heterogeneity

Given the strictly defined inclusion criteria for studies (diagnosis/condition, loading intervention and outcome measure), we anticipate clinical heterogeneity will be limited.

A chi-square test will evaluate the statistical significance of heterogeneity. The *I*^2^ statistic will estimate the amount of study heterogeneity based on the *p* value being ≤0.10 or the *I*^2^ value being ≥40% as suggested in Part 2, Section 9.5.2 of the Cochrane Handbook for Systematic Reviews of Interventions [22].

Where substantial heterogeneity (*P* ≤ 0.10 or *I*^2^ ≥ 40%) is found a sub-group analysis investigating the possible impact of a study will be determined by completing a sensitivity analysis. This analysis will involve an exclusion of pre-determined subgroups from heterogeneity analysis. Using the aforementioned statistical tests, the heterogeneity of the remaining studies will be determined. The following sub-groups will be analysed for their effect on heterogeneity:

- Studies in which the standard deviation was inputted as per the methods section above.
- Studies in which the adherence was not reported.
- Studies which used different exercise protocols as the comparator to heavy eccentric calf training.
- Studies in which both heavy eccentric calf training and the exercise intervention used as the comparator both received placebo interventions.
- Studies in which there was a high-risk of bias as assessed by the RoB 2.0 tool.

#### Assessment of reporting biases

The possible influence of publication/ small study biases on review findings will be considered. The influence of small study biases will be addressed by the risk of bias criterion ‘study size’. Studies with sample sizes less than 50 will be considered as representing high risk of small sample bias, studies with samples between 50 and 200 will be classified as moderate risk of small sample bias and studies with sample sizes greater than 200 will be classed as low risk of small sample bias [23].

Funnel plots will be visually inspected to explore the likelihood of reporting biases when at least 10 studies are included in a meta-analysis for a specific follow-up time point and included studies differ in size. For continuous outcomes, the Egger’s test [24] will be used to detect possible small study bias as recommended in Part 2, Section 10.4.3.1 of the Cochrane Handbook for Systematic Reviews of Interventions [22].

#### Assessment of the quality of the body of evidence

Assessment of the quality of the body of evidence was assessed using the GRADE approach [25] as recommended in Part 2, Section 12.2.1 of the Cochrane Handbook for Systematic Reviews of Interventions [22]. The GRADE approach involves making an overall judgement on the quality of the body of evidence based on the overall risk of bias, consistency of results, directness of the evidence and publication bias [25].

#### Data synthesis

Meta-analysis will be performed using an inverse variance statistical method and random effects analysis model in Review Manager version 5.3 (Review Manage [Computer program] version 5.3. Copenhagen: The Nordic Cochrane Centre, The Cochrane Collaboration, 2014.) to calculate the mean difference. Precise comparisons will be determined and presented from the included studies. Comparisons of interest are as follows:

- Heavy eccentric calf training versus wait-and-see
- Heavy eccentric calf training versus sham exercise
- Heavy eccentric calf training versus traditional physiotherapy
- Heavy eccentric calf training versus different exercise protocols

#### Sensitivity analysis

Sensitivity analysis will be conducted by excluding a sub-group of studies to assess their influence on the overall effect size and measures of heterogeneity. In Review Manager version 5.3, this is completed by allocating a weight of 0% to the study, meaning it is excluded from any analyses. A sensitivity analysis will be conducted on the following subgroups:

- Studies in which the standard deviation was inputted as per the methods section above.
- Studies in which the adherence was not reported.
- Studies which used different exercise protocols as the comparator to heavy eccentric calf training.
- Studies in which both heavy eccentric calf training and the exercise intervention used as the comparator both received placebo interventions.
- Studies in which there was a high-risk of bias as assessed by the RoB 2.0 tool.

#### Systematic review registration

This protocol has been registered on the PROSPERO CRD4201804493.

## Discussion

Heavy eccentric calf training is currently advocated for as the gold standard for treatment of mid-portion Achilles tendinopathy. However, to date, no systematic has compared this intervention to wait-and-see, sham rehabilitation, traditional physiotherapy or different exercise rehabilitation protocols. This systematic review aims to complete a comprehensive systematic review and meta-analysis of the current gold standard of treatment in mid-portion Achilles tendinopathy to determine if it really is the gold standard of management for mid-portion Achilles tendinopathy.

### Reporting standards

This systematic review protocol was written as per the PRISMA-P guidelines and the checklist is attached (Additional file 2).

## Additional files

Additional file 1: The RoB 2.0 tool (individually randomised, parallel group trials). (PDF 862 kb)

Additional file 2: PRISMA-P 2015 Checklist. (DOCX 30 kb)

## Abbreviations

AT: Achilles tendinopathy
BMI: Body mass index
GRADE: The Grade of Recommendation, Assessment, Development and Evaluation
PRISMA: Preferred Reporting Items for Systematic Reviews and Meta-Analyses
RoB: Risk of bias
SD: Standard deviation
VISA-A: Victorian Institute of Sports Assessment-Achilles
