# Supplementary material for: Is heavy eccentric calf training superior to wait-and-see, sham rehabilitation, traditional physiotherapy and other exercise interventions for pain and function in mid-portion Achilles tendinopathy?
Source: Syst Rev. 2018 Apr 13;7:58. doi: 10.1186/s13643-018-0725-6 (PMC5899347; doi:10.1186/s13643-018-0725-6)
Supplement: Supplementary file 1 — The RoB 2.0 tool (individually randomised, parallel group trials). (PDF 862 kb) [file 13643_2018_725_MOESM1_ESM.pdf]

The RoB 2.0 tool (individually randomized, parallel group trials)

Study design

- ☑ Randomized parallel group trial
- ☐ Cluster-randomized trial
- ☐ Randomized cross-over or other matched design

**Specify which outcome is being assessed for risk of bias**	
**Specify the numerical result being assessed.** In case of multiple alternative analyses being presented, specify the numeric result (e.g. RR = 1.52 (95% CI 0.83 to 2.77) and/or a reference (e.g. to a table, figure or paragraph) that uniquely defines the result being assessed.	

Is your aim for this study…?

- ☐ to assess the effect of *assignment to intervention*
- ☐ to assess the effect of *starting and adhering to intervention*

Which of the following sources have you obtained to help inform your risk of bias judgements (tick as many as apply)?

- ☐ Journal article(s) with results of the trial
- ☐ Trial protocol
- ☐ Statistical analysis plan (SAP)
- ☐ Non-commercial trial registry record (e.g. ClinicalTrials.gov record)
- ☐ Company-owned trial registry record (e.g. GSK Clinical Study Register record)
- ☐ "Grey literature" (e.g. unpublished thesis)
- ☐ Conference abstract(s) about the trial
- ☐ Regulatory document (e.g. Clinical Study Report, Drug Approval Package)
- ☐ Research ethics application
- ☐ Grant database summary (e.g. NIH RePORTER, Research Councils UK Gateway to Research)
- ☐ Personal communication with trialist
- ☐ Personal communication with the sponsor

© 2016 by the authors. This work is licensed under a Creative Commons Attribution-NonCommercial-NoDerivatives 4.0 International License.1

Risk of bias assessment

Responses underlined in green are potential markers for low risk of bias, and responses in red are potential markers for a risk of bias. Where questions relate only to sign posts to other questions, no formatting is used.

Bias domain	Signalling questions	Elaboration	Response options
**Bias arising from the randomization process**	1.1 Was the allocation sequence random?	"Yes" if a random component was used in the sequence generation process such as using a computer generated random numbers, referring to a random number table, minimization, coin tossing; shuffling cards or envelopes; throwing dice; or drawing of lots. Minimization may be implemented without a random element, and this is considered to be equivalent to being random.<br>"No" if the sequence is non-random, such that it is either likely to introduce confounding, or is predictable or difficult to conceal, e.g. alternation, methods based on dates (of birth or admission) or patient record numbers, allocation decision made by clinicians or participants, based on the availability of the intervention, or any other systematic or haphazard method.<br>If the only information about randomization methods is to state that the study is randomized, then this signalling question should generally be answered as "No information". There may be situations in which a judgement is made to answer "Probably No" or "Probably yes". For example, if the study was large, conducted by an independent trials unit or carried out for regulatory purposes, then it may be reasonable to assume that the sequence was random. Alternatively, if other (contemporary) trials by the same investigator team have clearly used non-random sequences, it might be reasonable to assume that the current study was done using similar methods. Similarly, if participants and personnel are all unaware of intervention assignments throughout/during the trial (blinding or masking), this may be an indicator that the allocation process was also concealed, but this will not necessarily always be the case.<br>If the allocation sequence was clearly concealed but there is no information about how the sequence was generated, it will often be reasonable to assume that the sequence was random (although this will not necessarily always be the case).	Y / PY / PN / N / NI
	1.2 Was the allocation sequence concealed until participants were recruited and assigned to interventions?	"Yes" if any form of remote or centrally administered randomization, where the process of allocation is controlled by an outsourced unit or organization, independent of the enrolment personnel (e.g. independent central pharmacy, telephone or internet-based randomization service providers).	Y / PY / PN / N / NI

© 2016 by the authors. This work is licensed under a Creative Commons Attribution-NonCommercial-NoDerivatives 4.0 International License.

		"Yes" if envelopes or drug containers were used appropriately. Envelopes should be sequentially numbered, sealed with a tamper proof seal and opaque. Drug containers should be sequentially numbered and of identical appearance. This level of detail is rarely provided in reports, and a judgement may be required (e.g. "Probably yes" or "Probably no").<br>"No" if there is reason to suspect the enrolling investigator or the participant had knowledge of the forthcoming allocation.	
	1.3 Were there baseline imbalances that suggest a problem with the randomization process?	*NB Imbalances that are small and compatible with chance should not be highlighted using the RoB 2.0 tool; chance imbalances are not bias.*<br>Answer "No" if no imbalances are apparent or if any observed imbalances are compatible with chance<br>Answer "Yes" if there are imbalances that indicate problems with the randomization process, including:<br>(1) unusually large differences between intervention group sizes; or<br>(2) a substantial excess in statistically significant differences in baseline characteristics than would be expected by chance alone; or<br>(3) imbalance in key prognostic factors (or baseline measures of outcome variables) that are unlikely to be due to chance.<br>An answer of "Yes/Probably yes" may exceptionally be given if the groups are surprisingly balanced in a way that appears incompatible with chance and the randomization methods, thus raising suspicion about the methods used.<br>In some circumstances, it may be reasonable to answer "Yes/Probably yes" (rather than "No information") when there is a surprising lack of information on baseline characteristics when such information could reasonably be expected to be available/reported.<br>Answer "No information" when there is no *useful* baseline information available (e.g. abstracts, or studies that reported only baseline characteristics of participants in the final analysis).<br>The answer to this question should not be used to influence answers to questions 1.1 or 1.2. For example, if the trial has large baseline imbalances, but authors report adequate randomization methods, questions 1.1 and 1.2 should still be answered on the basis of the reported adequate methods, and any concerns about the imbalance	Y / PY / PN / N / NI

© 2016 by the authors. This work is licensed under a Creative Commons Attribution-NonCommercial-NoDerivatives 4.0 International License.

		should be raised in the answer to the question 1.3 and reflected in the domain-level risk of bias judgement).	
	**Risk of bias judgement**	See Figure 1.	Low / High / Some concerns
	Optional: What is the predicted direction of bias arising from the randomization process?	If the likely direction of bias can be predicted, it is helpful to state this. The direction might be characterized either as being towards (or away from) the null, or as being in favour of one of the interventions.	Favours experimental / Favours comparator / Towards null /Away from null / Unpredictable

© 2016 by the authors. This work is licensed under a Creative Commons Attribution-NonCommercial-NoDerivatives 4.0 International License.

Bias domain	Signalling questions	Elaboration	Response options
**Bias due to deviations from intended interventions**	**If your aim for this study is to assess the effect of assignment to intervention, answer the following questions**		
	2.1. Were participants aware of their assigned intervention during the trial?	If participants are aware of their group assignment, it is more likely that additional health-related behaviours will differ between the assigned intervention groups, so risk of bias will be higher. Masking participants, which is most commonly achieved through use of a placebo or sham intervention, may prevent such differences.	Y / PY / PN / N / NI
	2.2. Were carers and trial personnel aware of participants' assigned intervention during the trial?	If those involved in caring for participants or making decisions about their health care are aware of the assigned intervention, then implementation of the intended intervention, or administration of additional co-interventions, may differ between the assigned intervention groups. Masking carers and trial personnel, which is most commonly achieved through use of a placebo, may prevent such differences.	Y / PY / PN / N / NI
	2.3. If Y/PY/NI to 2.1 or 2.2: Were there deviations from the intended intervention beyond what would be expected in usual practice?	When interest focusses on the effect of assignment to intervention, it is important to distinguish between:<br>(a) deviations that happen in usual practice following the intervention and so are part of the intended intervention (for example, cessation of a drug intervention because of acute toxicity); and<br>(b) deviations from intended intervention that arise due to expectations of a difference between intervention and comparator (for example because participants feel "unlucky" to have been assigned to the comparator group and therefore seek the active intervention, or components of it, or other interventions).<br>We use the term "usual practice" to refer to the usual course of events in a non-trial context. Because deviations that arise due to expectations of a difference between intervention and comparator are not part of usual practice, they may lead to biased effect estimates that do not reflect what would happen to participants assigned to the interventions in practice.<br>Trialists do not always report (and do not necessarily know) whether deviations that are not part of usual practice actually occurred. Therefore the answer "No information" may be appropriate. However, if such deviations *probably* occurred you should answer "Probably yes".	NA / Y / PY / PN / N / NI
	2.4. If Y/PY to 2.3: Were these deviations from intended intervention unbalanced between groups *and* likely to have affected the outcome?	Deviations from intended interventions that do not reflect usual practice will be important if they affect the outcome, but not otherwise. Furthermore, bias will arise only if there is imbalance in the deviations across the two groups.	NA / Y / PY / PN / N / NI

© 2016 by the authors. This work is licensed under a Creative Commons Attribution-NonCommercial-NoDerivatives 4.0 International License.

	2.5 Were any participants analysed in a group different from the one to which they were assigned?	This question addresses one of the fundamental aspects of an "intention-to-treat" approach to the trial analysis: that participants are analysed in the groups to which they were assigned through randomization. If some participants did not receive their assigned intervention, and such participants were analysed according to intervention received, then the balance between intervention groups created by randomization is lost.	Y / PY / PN / N / NI
	2.6 If Y/PY/NI to 2.5: Was there potential for a substantial impact (on the estimated effect of intervention) of analysing participants in the wrong group?	Risk of bias will be high in a randomized trial in which sufficiently many participants were analysed in the wrong intervention group that there could have been a substantial impact on the results. There is potential for a substantial impact if more than 5% of participants were analysed in the wrong group, but for rare events there could be an impact for a smaller proportion.	NA / Y / PY / PN / N / NI
	**If your aim for this study is to assess the effect of starting and adhering to intervention, answer the following questions**		
	2.1. Were participants aware of their assigned intervention during the trial?	If participants are aware of their group assignment, it is more likely that additional health-related behaviours will differ between the intervention groups, so risk of bias will be higher. Masking participants, which is most commonly achieved through use of a placebo, may prevent such differences.	Y / PY / PN / N / NI
	2.2. Were carers and trial personnel aware of participants' assigned intervention during the trial?	If those involved in caring for participants and those otherwise involved in the trial are aware of group assignment, then it is more likely that implementation of the intended intervention, or the administration of additional co-interventions, will differ between the intervention groups. Masking carers and trial personnel, which is most commonly achieved through use of a placebo, may prevent such differences.	Y / PY / PN / N / NI
	2.3. If Y/PY/NI to 2.1 or 2.2: Were important co-interventions balanced across intervention groups?	Risk of bias will be higher if unplanned co-interventions were implemented in a way that would bias the estimated effect of intervention. Co-interventions will be important if they affect the outcome, but not otherwise. Bias will arise only if there is imbalance in such co-interventions between the intervention groups. Consider the co-interventions, including any pre-specified co-interventions, that are likely to affect the outcome and to have been administered in this study. Consider whether these co-interventions are balanced between intervention groups.	NA / Y / PY / PN / N / NI
	2.4. Was the intervention implemented successfully?	Risk of bias will be higher if the intervention was not implemented as intended by, for example, the health care professionals delivering care during the trial. Consider whether implementation of the intervention was successful for most participants.	Y / PY / PN / N / NI
	2.5. Did study participants adhere to the assigned intervention regimen?	Risk of bias will be higher if participants did not adhere to the intervention as intended. Lack of adherence includes imperfect compliance, cessation of intervention, crossovers to the comparator intervention and switches to another active intervention. Consider available information on the proportion of study participants who continued with their	Y / PY / PN / N / NI

© 2016 by the authors. This work is licensed under a Creative Commons Attribution-NonCommercial-NoDerivatives 4.0 International License.

		assigned intervention throughout follow up, and answer "No" or "Probably No" if this proportion is high enough to raise concerns. Answer "Yes" for studies of interventions that are administered once, so that imperfect adherence is not possible.	
	2.6. If N/PN/NI to 2.3, 2.4 or 2.5: Was an appropriate analysis used to estimate the effect of starting and adhering to the intervention?	It is possible to conduct an analysis that corrects for some types of deviation from the intended intervention. Examples of appropriate analysis strategies include inverse probability weighting or instrumental variable estimation. It is possible that a paper reports such an analysis without reporting information on the deviations from intended intervention, but it would be hard to judge such an analysis to be appropriate in the absence of such information.<br>If everyone in one group received a co-intervention, adjustments cannot be made to overcome this.<br>Some examples of analysis strategies that would not be appropriate to estimate the effect of intended intervention are (i) "ITT analysis", (ii) "per protocol analysis", and (iii) "analysis by treatment received".	NA / Y / PY / PN / N / NI
	**Risk of bias judgement**	See Figure 2 and Figure 3.	Low / High / Some concerns
	Optional: What is the predicted direction of bias due to deviations from intended interventions?	If the likely direction of bias can be predicted, it is helpful to state this. The direction might be characterized either as being towards (or away from) the null, or as being in favour of one of the interventions.	Favours experimental / Favours comparator / Towards null /Away from null / Unpredictable

© 2016 by the authors. This work is licensed under a Creative Commons Attribution-NonCommercial-NoDerivatives 4.0 International License.

Bias domain	Signalling questions	Elaboration	Response options
**Bias due to missing outcome data**	3.1 Were outcome data available for all, or nearly all, participants randomized?	The appropriate study population for an analysis of the intention to treat effect is all randomized patients.<br>Note that imputed data should be regarded as missing data, and not considered as "outcome data" in the context of this question.<br>"Nearly all" (equivalently, a low or modest amount of missing data) should be interpreted as "enough to be confident of the findings", and a suitable proportion depends on the context.<br>For continuous outcomes, availability of data from 95% (or possibly 90%) of the participants would often be sufficient. For dichotomous outcomes, the proportion required is directly linked to the risk of the event. If the observed number of events is much greater than the number of participants with missing outcome data, the bias would necessarily be small.	Y / PY / PN / N / NI
	3.2 If N/PN/NI to 3.1: Are the proportions of missing outcome data and reasons for missing outcome data similar across intervention groups?	"Similar" (with regard to proportion and reasons for missing outcome data) includes some minor degree of discrepancy across intervention groups as expected by chance. Assessment of comparability of reasons for missingness requires the reasons to be reported.	NA / Y / PY / PN / N / NI
	3.3 If N/PN/NI to 3.1: Is there evidence that results were robust to the presence of missing outcome data?	Evidence for robustness may come from how missing data were handled in the analysis and whether sensitivity analyses were performed by the trial investigators, or from additional analyses performed by the systematic reviewers.	NA / Y / PY / PN / N / NI
	**Risk of bias judgement**	See Figure 4	Low / High / Some concerns
	Optional: What is the predicted direction of bias due to missing outcome data?	If the likely direction of bias can be predicted, it is helpful to state this. The direction might be characterized either as being towards (or away from) the null, or as being in favour of one of the interventions.	Favours experimental / Favours comparator / Towards null /Away from null / Unpredictable

© 2016 by the authors. This work is licensed under a Creative Commons Attribution-NonCommercial-NoDerivatives 4.0 International License.

Bias domain	Signalling questions	Elaboration	Response options
**Bias in measurement of the outcome**	4.1 Were outcome assessors aware of the intervention received by study participants?	“No” if outcome assessors were blinded to intervention status. In studies where participants report their outcomes themselves (i.e., participant-reported outcome), the outcome assessor is the study participant.	Y / PY / PN / N / NI
	4.2 If Y/PY/NI to 4.1: Was the assessment of the outcome likely to be influenced by knowledge of intervention received?	Knowledge of the assigned intervention may impact on participant-reported outcomes (such as level of pain), observer-reported outcomes involving some judgement, and intervention provider decision outcomes, while not impacting on other outcomes such as observer reported outcomes not involving judgement such as all-cause mortality. In many circumstances the assessment of *observer reported outcomes not involving judgement* such as all-cause mortality might be considered to be unbiased, even if outcome assessors were aware of intervention assignments.	NA / Y / PY / PN / N / NI
	**Risk of bias judgement**	See Figure 5.	Low / High / Some concerns
	Optional: What is the predicted direction of bias due to measurement of the outcome?	If the likely direction of bias can be predicted, it is helpful to state this. The direction might be characterized either as being towards (or away from) the null, or as being in favour of one of the interventions.	Favours experimental / Favours comparator / Towards null /Away from null / Unpredictable

© 2016 by the authors. This work is licensed under a Creative Commons Attribution-NonCommercial-NoDerivatives 4.0 International License.

Bias domain	Signalling questions	Elaboration	Response options
**Bias in selection of the reported result**	Are the reported outcome data likely to have been selected, on the basis of the results, from...		
	5.1. ... multiple outcome measurements (e.g. scales, definitions, time points) within the outcome domain?	A particular outcome domain (i.e. a true state or endpoint of interest) may be **measured** in multiple ways. For example, the domain pain may be measured using multiple scales (e.g. a visual analogue scale and the McGill Pain Questionnaire), each at multiple time points (e.g. 3, 6 and 12 weeks post-treatment). If multiple measurements were made, but only one or a subset is reported on the basis of the results (e.g. statistical significance), there is a high risk of bias in the fully reported result.<br>**A response of "Yes/Probably yes" is reasonable if:**<br>There is clear evidence (usually through examination of a trial protocol or statistical analysis plan) that a domain was measured in multiple ways, but data for only one or a subset of measures is fully reported (without justification), and the fully reported result is likely to have been selected on the basis of the results. Selection on the basis of the results arises from a desire for findings to be newsworthy, sufficiently noteworthy to merit publication, or to confirm a prior hypothesis. For example, trialists who have a preconception or vested interest in showing that an experimental intervention is beneficial may be inclined to selectively report outcome measurements that are favourable to the experimental intervention.<br>**A response of "No/Probably no" is reasonable if:**<br>There is clear evidence (usually through examination of a trial protocol or statistical analysis plan) that all reported results for the outcome domain correspond to all intended outcome measurements.<br>or<br>There is only one possible way in which the outcome domain can be measured (hence there is no opportunity to select from multiple measures).<br>or<br>Outcome measurements are inconsistent across different reports on the same trial, but the trialists have provided the reason for the inconsistency and it is not related to the nature of the results.<br>**A response of "No information" is reasonable if:**	Y / PY / PN / N / NI

© 2016 by the authors. This work is licensed under a Creative Commons Attribution-NonCommercial-NoDerivatives 4.0 International License.

		Analysis intentions are not available, or the analysis intentions are not reported in sufficient detail to enable an assessment, and there is more than one way in which the outcome domain could have been measured.	
	5.2 ... multiple analyses of the data?	A particular outcome domain may be **analysed** in multiple ways. Examples include: unadjusted and adjusted models; final value *vs* change from baseline *vs* analysis of covariance; transformations of variables; conversion of continuously scaled outcome to categorical data with different cut-points; different sets of covariates for adjustment; different strategies for dealing with missing data. Application of multiple methods generates multiple effect estimates for a specific outcome domain. If multiple estimates are generated but only one or a subset is reported on the basis of the results (e.g. statistical significance), there is a high risk of bias in the fully reported result.<br>**A response of "Yes/Probably yes" is reasonable if:**<br>There is clear evidence (usually through examination of a trial protocol or statistical analysis plan) that a domain was analysed in multiple ways, but data for only one or a subset of analyses is fully reported (without justification), and the fully reported result is likely to have been selected on the basis of the results. Selection on the basis of the results arises from a desire for findings to be newsworthy, sufficiently noteworthy to merit publication, or to confirm a prior hypothesis. For example, trialists who have a preconception or vested interest in showing that an experimental intervention is beneficial may be inclined to selectively report analyses that are favourable to the experimental intervention.<br>**A response of "No/Probably no" is reasonable if:**<br>There is clear evidence (usually through examination of a trial protocol or statistical analysis plan) that all reported results for the outcome domain correspond to all intended analyses.<br>or<br>There is only one possible way in which the outcome domain can be analysed (hence there is no opportunity to select from multiple analyses).<br>or<br>Analyses are inconsistent across different reports on the same trial, but the trialists have provided the reason for the inconsistency and it is not related to the nature of the results.	Y / PY / PN / N / NI

© 2016 by the authors. This work is licensed under a Creative Commons Attribution-NonCommercial-NoDerivatives 4.0 International License.

		**A response of "No information" is reasonable if:**<br>Analysis intentions are not available, or the analysis intentions are not reported in sufficient detail to enable an assessment, and there is more than one way in which the outcome domain could have been analysed.	
	**Risk of bias judgement**	See Figure 6.	Low / High / Some concerns
	Optional: What is the predicted direction of bias due to selection of the reported result?	If the likely direction of bias can be predicted, it is helpful to state this. The direction might be characterized either as being towards (or away from) the null, or as being in favour of one of the interventions.	Favours experimental / Favours comparator / Towards null /Away from null / Unpredictable

© 2016 by the authors. This work is licensed under a Creative Commons Attribution-NonCommercial-NoDerivatives 4.0 International License.

Bias domain	Signalling questions	Elaboration	Response options
**Overall bias**	**Risk of bias judgement**	See Table 1	Low / High / Some concerns
	Optional:<br>What is the overall predicted direction of bias for this outcome?	If the likely direction of bias can be predicted, it is helpful to state this. The direction might be characterized either as being towards (or away from) the null, or as being in favour of one of the interventions.	Favours experimental / Favours comparator / Towards null /Away from null / Unpredictable

This work is licensed under a Creative Commons Attribution-NonCommercial-NoDerivatives 4.0 International License.

Figure 1. **Suggested algorithm for reaching risk of bias judgements for bias arising from the randomization process. (*In some cases a judgement of "High risk" would be appropriate.). This is only a suggested decision tree: all default judgements can be overridden by assessors.**

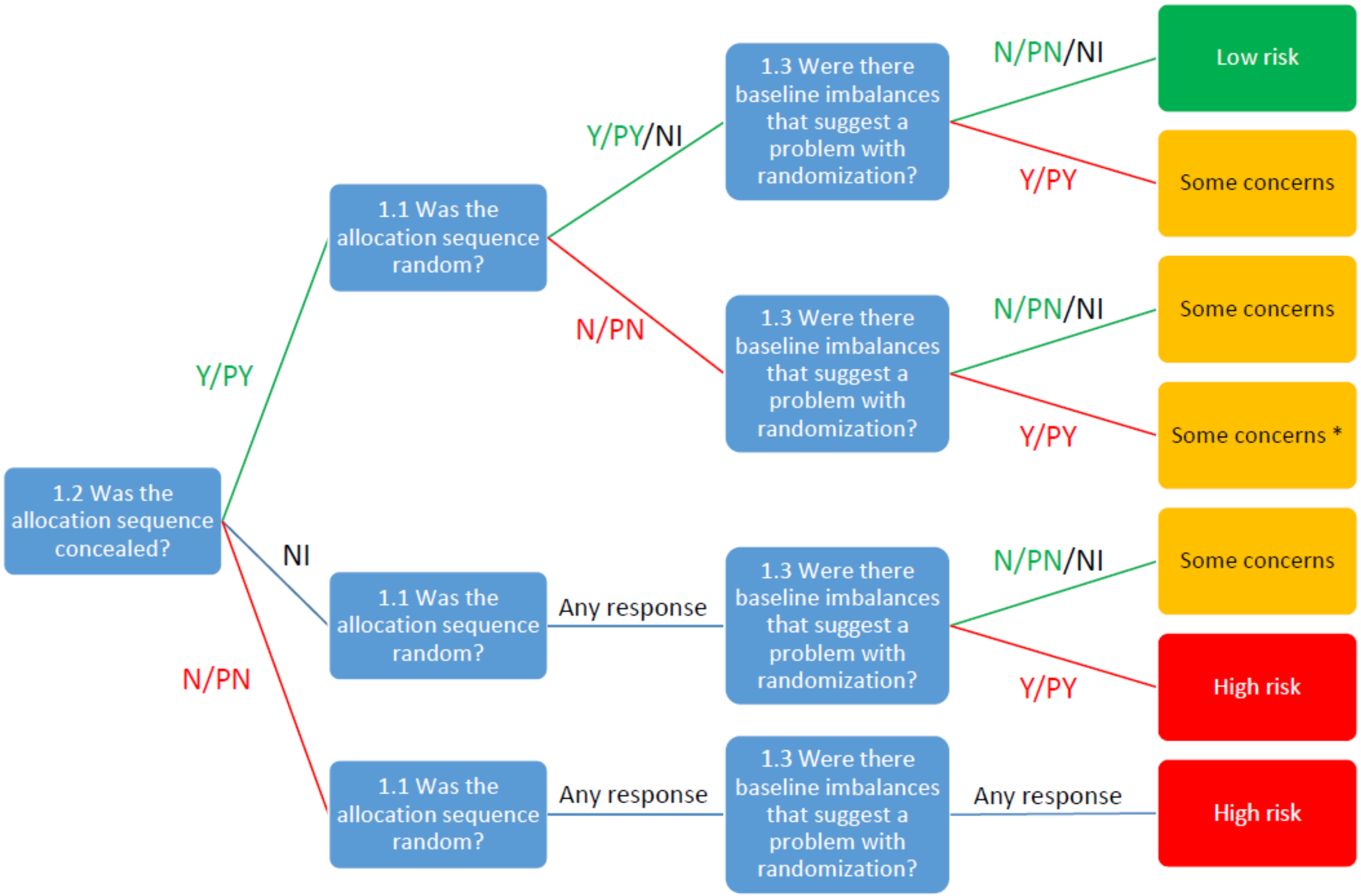

© 2016 by the authors. This work is licensed under a Creative Commons Attribution-NonCommercial-NoDerivatives 4.0 International License.

Figure 2. Suggested algorithm for reaching risk of bias judgements for bias due to deviations from intended interventions (*effect of assignment to intervention*). This is only a suggested decision tree: all default judgements can be overridden by assessors.

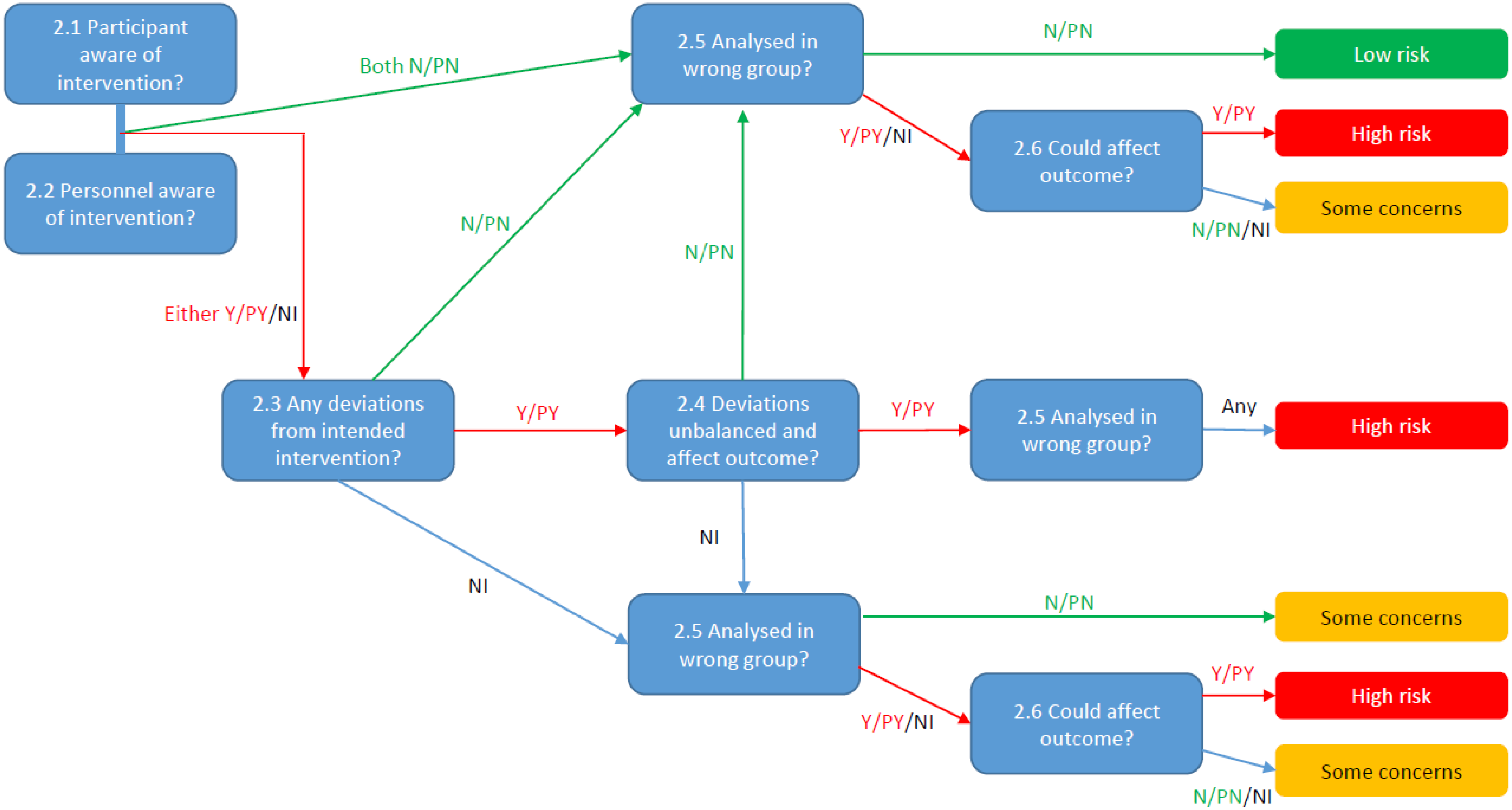

© 2016 by the authors. This work is licensed under a Creative Commons Attribution-NonCommercial-NoDerivatives 4.0 International License.

Figure 3. Suggested algorithm for reaching risk of bias judgements for bias due to deviations from intended interventions (*effect starting and adhering to intervention)*. This is only a suggested decision tree: all default judgements can be overridden by assessors.

© 2016 by the authors. This work is licensed under a Creative Commons Attribution-NonCommercial-NoDerivatives 4.0 International License.

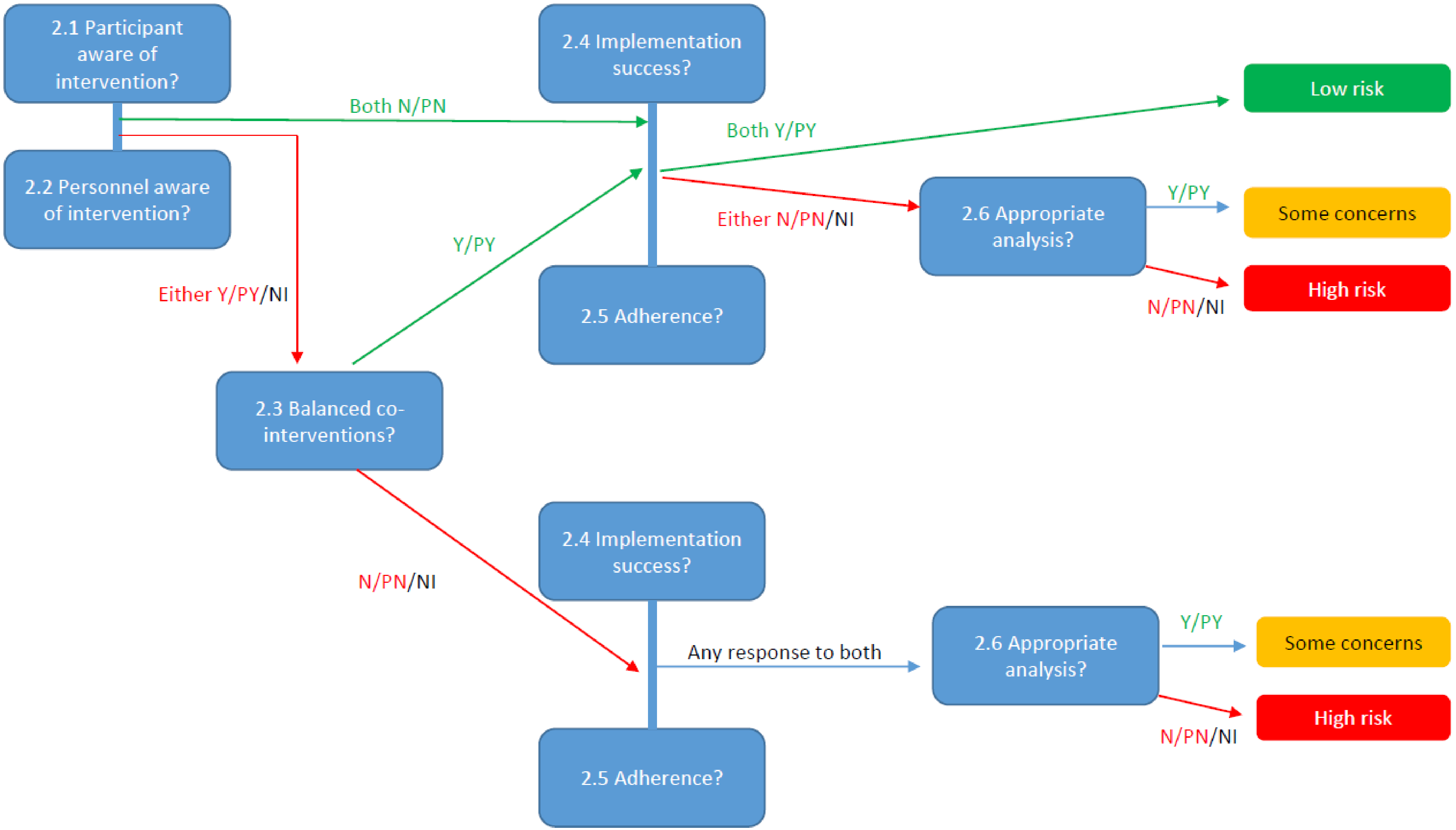

© 2016 by the authors. This work is licensed under a Creative Commons Attribution-NonCommercial-NoDerivatives 4.0 International License.

Figure 4. Suggested algorithm for reaching risk of bias judgements for bias due to missing outcome data. This is only a suggested decision tree: all default judgements can be overridden by assessors

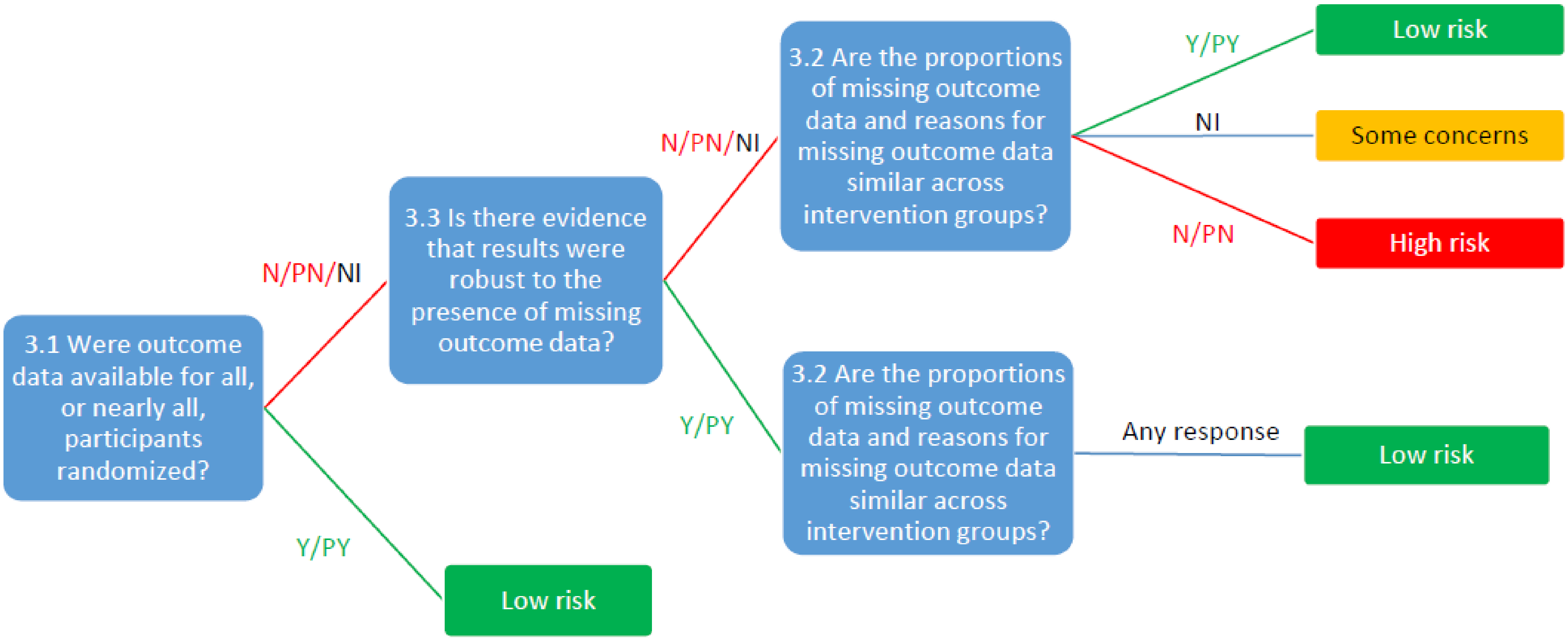

© 2016 by the authors. This work is licensed under a Creative Commons Attribution-NonCommercial-NoDerivatives 4.0 International License.

Figure 5. Suggested algorithm for reaching risk of bias judgements for bias in measurement of the outcome. This is only a suggested decision tree: all default judgements can be overridden by assessors.

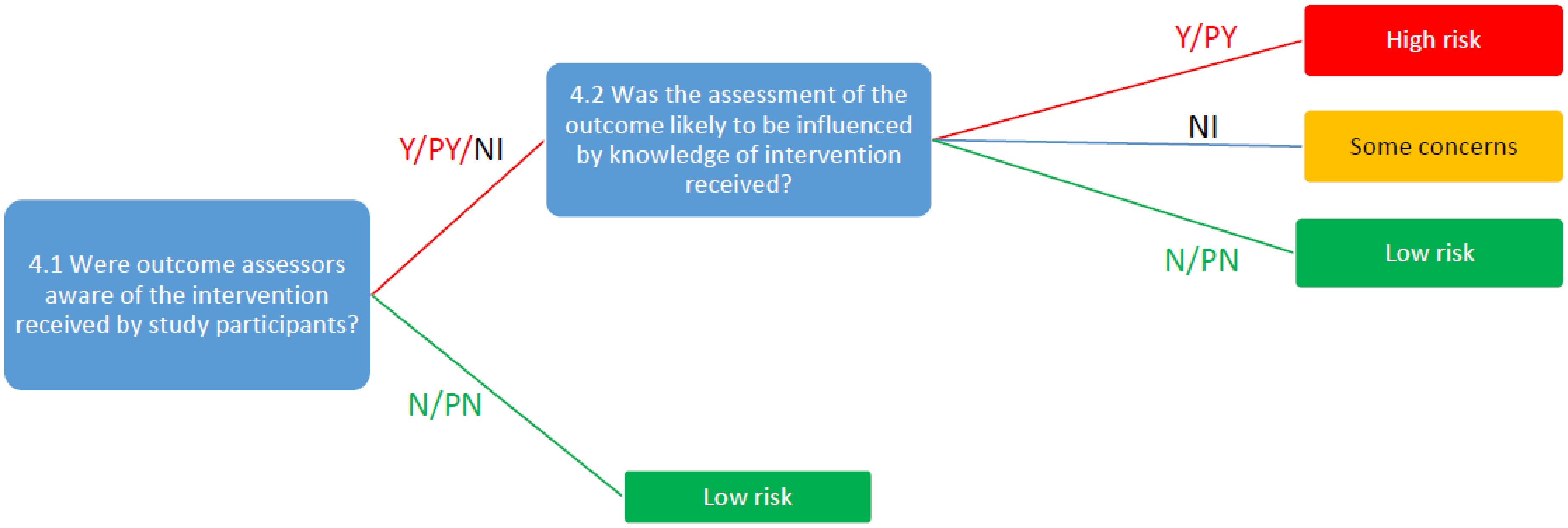

© 2016 by the authors. This work is licensed under a Creative Commons Attribution-NonCommercial-NoDerivatives 4.0 International License.

Figure 6. Suggested algorithm for reaching risk of bias judgements for bias in selection of the reported result. This is only a suggested decision tree: all default judgements can be overridden by assessors

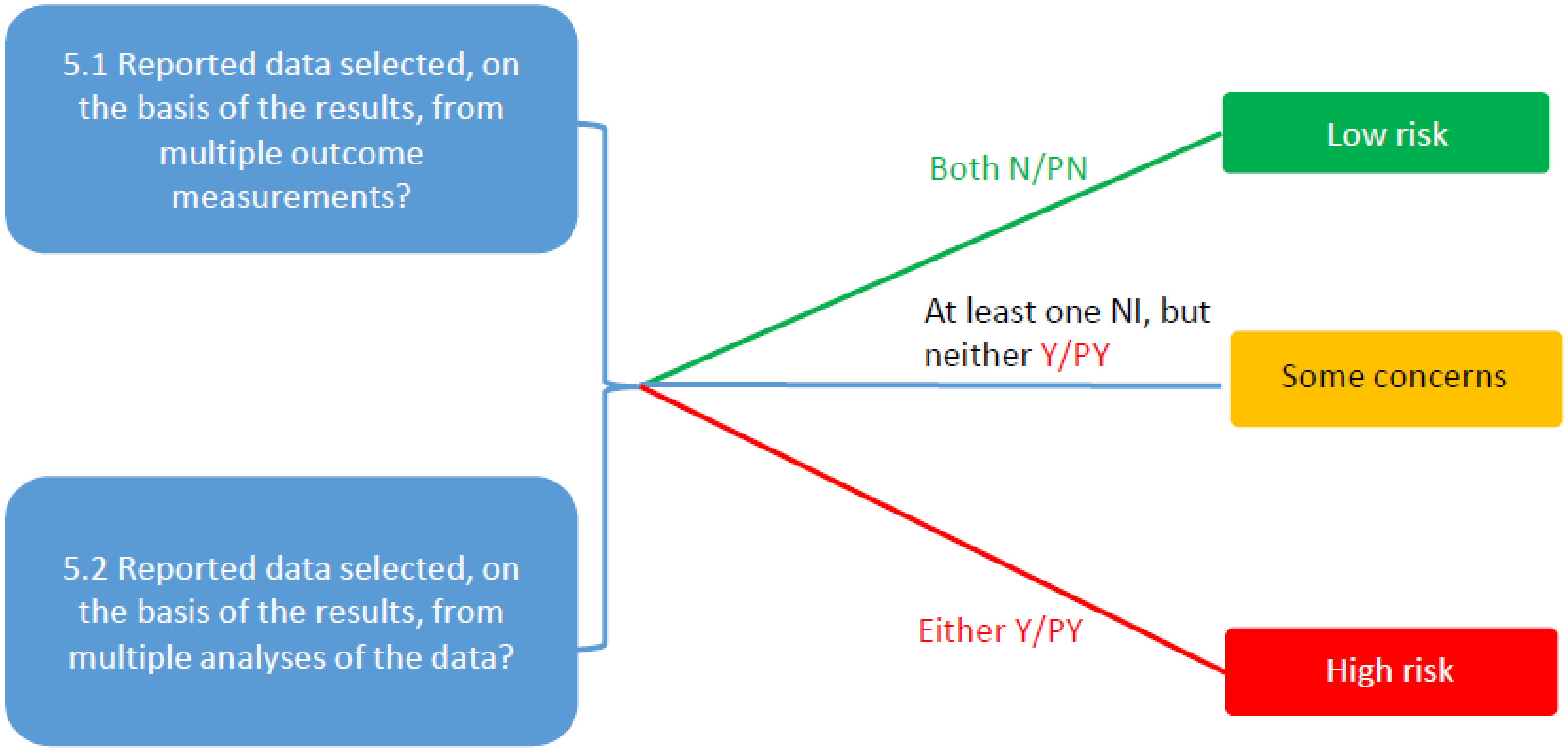

© 2016 by the authors. This work is licensed under a Creative Commons Attribution-NonCommercial-NoDerivatives 4.0 International License.

Table 1. Reaching an overall risk of bias judgement for a specific outcome.

Overall risk of bias judgement	Criteria
Low risk of bias	The study is judged to be at **low risk of bias for all domains** for this result.
Some concerns	The study is judged to be at **some concerns** in at least one domain for this result.
High risk of bias	The study is judged to be at **high risk of bias** in at least one domain for this result.<br>Or<br>The study is judged to have **some concerns** for **multiple domains** in a way that substantially lowers confidence in the result.

© 2016 by the authors. This work is licensed under a Creative Commons Attribution-NonCommercial-NoDerivatives 4.0 International License.
